# Editorial: Brain Stimulation and Behavioral Change

**DOI:** 10.3389/fnbeh.2019.00020

**Published:** 2019-02-08

**Authors:** Claudio Lucchiari, Nicholas J. Kelley, Maria Elide Vanutelli, Roberta Ferrucci

**Affiliations:** ^1^Department of Philosophy, Università degli Studi di Milano, Milan, Italy; ^2^Department of Psychology, Northwestern University, Evanston, IL, United States; ^3^Fondazione Ca' Granda, IRCCS Ospedale Maggiore Policlinico, Milan, Italy; ^4^“Aldo Ravelli” Research Center for Neurotechnology and Experimental Brain Therapeutics, University of Milan Medical School, Milan, Italy

**Keywords:** brain stimulation, tDCS, TMS, behavioral change, neuro-cognitive correlates

The use of brain stimulation techniques has recently exploded. Certainly, one reason for this explosion of research is that it is a cheap way to change behavior. However, on the other hand we still know very little about the underlying neural mechanisms. In the research topic “Brain stimulation and behavioral change” we highlight both empirical research and theoretical reviews demonstrating the ability of non-invasive brain stimulation techniques to affect behavior change in a variety of domains including: psychiatric symptoms (e.g., anorexia, psychosis), neurological rehabilitation, motor control, visual perception, self-regulation, and social cognition. This research topic highlights the unique opportunity brain stimulation provides for understanding neuro-functional brain networks, testing theoretical constructs, assessing the presence of cognitive deficits or potentials, mitigating symptoms of disease, and promoting health.

Along this path, heterogeneous by its nature but also rich in suggestions and unexpected connections, Ferrucci et al. described a transcranial direct current stimulation (tDCS) study on patients with fronto-temporal dementia. The authors, by using a stimulation protocol of 5 consecutive days, found an improvement in the visuo-attentive abilities of the patients following an anodal stimulation applied over the fronto-temporal cortex after 1 month from the end of the treatment, as well as a short-term improvement of some neuropsychiatric symptoms. Although the study needs further developments on larger samples, it is worth noting the correlation found between the decrease of the frontal slow waves and the cognitive improvement of the patients. This finding confirms that transcranial stimulation can target not only a specific symptom but also neuro-functional correlations, thus allowing a highly focused evidence-based treatment with a limited impact on the patient's quality of life.

Similarly, the study performed by Bocci et al. in patients with amblyopia (strabismus) shows how the cathodal application of tDCS on the primary visual cortex contralateral to the defective eye reduces transcallosal inhibition as measured through visual evoked potentials (VEPs) and compared to that of healthy subjects, thus allowing an increase in visual acuity. In this case, the study not only allows us to hypothesize future treatments targeted at adult patients, but it also permits to support the hypothesis of the role of the interhemispheric balance in the physiopathology of amblyopia.

In the same direction goes the review proposed by Gupta et al. which is aimed at summarizing the literature about the potential of transcranial stimulation to improve the symptoms and quality of life of psychotic patients. Neuropsychiatric contexts are increasingly benefiting from transcranial stimulation applications in support to the limited effects of pharmacotherapy and its side effects. The review shows how tDCS can be used safely and profitably both to mitigate positive psychiatric symptoms (hallucinations and delusions) and to enhance cognitive functioning.

The experiment conducted by the group of Costanzo et al. also shows how tDCS can be a useful ally of the psychiatrist. In particular, the study shows the positive effect of a tDCS protocol in patients with anorexia nervosa (AN). The protocol, in fact, has proven to be superior to standard treatments in promoting food intake and stabilizing the weight of adolescents with AN. This finding is probably associated with the ability of tDCS to directly stimulate some nervous networks involved in the regulation of feeding and in reward and gratification which are usually difficult targets to reach with a standard intervention.

Still considering psychiatric applications, Dittert et al. showed how it is possible to apply tDCS to the ventromedial prefrontal cortex (vmPFC) to accelerate the extinction of conditioned learning. In this way, the authors investigated the use of a tDCS protocol with a large sample of healthy subjects in a controlled laboratory setting. However, the study also presents some critical aspects, since it was expected to increase the response to the stimulus but also to increase the reaction to the unconditioned one. This implies the necessity to better understand the mechanisms underlying vmPFC stimulation in conditioned learning before it can be applied in clinical settings.

The study by Behler et al. also shows critical aspects of tDCS application. In fact, the authors present a series of clinical cases with little significant effects in improving the symptoms associated with Tourette's syndrome.

In contrast, the protocol presented by Liu et al. based on transcranial magnetic stimulation (rTMS) of the cerebellum and the primary motor cortex (M1) proved to improve the motor symptoms of patients with Multiple System Atrophy. In particular, the improvement seems to be related to a normalization of the resting-state dynamics in the motor circuit (cerebellum-M1) targeted by rTMS, as demonstrated by fMRI. In this case, therefore, the intervention protocol has a very precise neuro-functional target that allows establishing a priori some experimentally verifiable hypotheses, both at a behavioral and neurophysiological level.

At this regard, it is particularly interesting the study proposed by Berger et al. that investigated the role of transcranial alternating current stimulation (tACS) on the oscillatory activity of subjects engaged in a bimanual coordination task by combining EEG and fNIRS. In this case, the authors demonstrated a specific neurophysiological effect (detectable by both EEG and fNIRS) of the tACS applied over the motor circuits involved in the chosen task within the parietal areas. Moreover, the choice of the experimental task proves to be increasingly relevant in evaluating the effect of specific transcranial stimulation protocols both in experimental and in applicative settings.

The review by Pixa and Pollok goes precisely in this direction. In fact, it describes the role of tDCS in enhancing motor learning, with reference to bimanual coordination tasks. The authors, in fact, report a generally positive impact of tDCS in promoting bimanual skills both in healthy subjects and patients with neurological disorders, showing the importance of using tDCS protocols where the stimulation is consistent with the motor tasks required.

However, transcranial stimulation, and particularly tDCS, can also be used at home to mitigate symptoms and improve the quality of life of patients with various diseases. In this way, remote treatment protocols can be hypothesized through the integration of stimulation procedures and e-health protocols, thus increasing patient compliance and reducing the costs of health institutions. In this sense, the study reported by Riggs et al. demonstrates the feasibility of remote-controlled tDCS protocols, as well as good patient compliance. This kind of applications will probably find wide diffusion in the near future.

Remaining in the field of non-traditional clinical applications of transcranial stimulation, Cancer and Antonietti showed how it is possible to design and implement tDCS protocols to improve some typical defects of learning disorders, such as reading speed. Stimulating the circuits involved in this process (left temporo-parietal cortex, right cerebellum, and left frontal cortices) improves the reading process (even if in a diversified way) both in adults and adolescents with dyslexia, but not in typical readers.

The study by Brunnauer et al. also aims to evaluate tDCS effects in non-traditional settings, such as the improvement of cognitive skills underlying driving skills. However, the study shows a poor impact of the left dorsolateral prefrontal cortex (DLPFC) stimulation on these skills.

The study by Wang et al. is similar to the previous one in terms of setting, but it is aimed at studying the neurophysiological mechanisms underlying gratitude. The authors used a tDCS protocol stimulating the mPFC during an economic game set in a working context. In the beginning, subjects were tested for their socio-cognitive skills. Then, they were required to pretend to be employees facing different choices. The cathodal stimulation over the mPFC proved to be capable of decreasing the effort of showing gratitude in employees with poor skills, while the anodal stimulation of the same area increased the effort in high-functioning employees. A further demonstration of how transcranial stimulation has differential effects according to the experimental setting but also according to the individual features.

Moreover, three reviews showed that transcranial stimulation can be used to study psychological constructs such as agency, goal-oriented behavior, and creativity. Specifically, Crivelli and Balconi have collected and discussed the literature related to the agent brain studied through transcranial stimulation, showing how this technique can be particularly useful in supporting neuroimaging data, as well as providing new lines of research. Also, the review by Kelley et al. discussed the findings obtained by stimulating the prefrontal cortices that may promote positive self-regulation. The principle is to alter the balance in the activity between the prefrontal cortex and the subcortical regions involved in emotion and reward processing.

Finally, the review by Lucchiari et al. summarized the existing literature on the promotion of creativity through tDCS. The critical discussion led to an explanatory model that correlates the stimulation of certain brain areas, such as the left lower frontal gyrus, and the balancing between the frontal cognitive control system and the default mode network. This balance can be considered the basis of the relationship between divergent and convergent thinking which transcranial stimulation can modify directly, thus promoting evident short-term effects.

In summary, the papers within this Research Topic suggest that the brain stimulation techniques play an important role in neuroscience, both as tools to improve our knowledge about the human mind, and to develop protocols to change dysfunctional behaviors, mitigate symptoms and improve cognitive and behavioral perfomance. However, results from original studies as well as from review articles highlight the importance of using specific and testable theoretical models about the neural circuits to be stimulated in order to improve the probability of success and prevent potential side effects before considering real-world applications.

## Author Contributions

All authors listed have made a substantial, direct and intellectual contribution to the work, and approved it for publication.

### Conflict of Interest Statement

The authors declare that the research was conducted in the absence of any commercial or financial relationships that could be construed as a potential conflict of interest.

